# The highly pathogenic H7N3 avian influenza strain from July 2012 in Mexico acquired an extended cleavage site through recombination with host 28S rRNA

**DOI:** 10.1186/1743-422X-10-139

**Published:** 2013-05-01

**Authors:** Sebastian Maurer-Stroh, Raphael TC Lee, Vithiagaran Gunalan, Frank Eisenhaber

**Affiliations:** 1 Bioinformatics Institute (BII), Agency for Science, Technology and Research (A*STAR), Singapore, Singapore; 2 National Public Health Laboratory, Communicable Diseases Division Ministry of Health, Singapore, Singapore; 3 School of Biological Sciences (SBS), Nanyang Technological University (NTU), Singapore, Singapore; 4 School of Computer Engineering (SCE), Nanyang Technological University (NTU), Singapore, Singapore; 5 Department of Biological Sciences (DBS), National University of Singapore (NUS), Singapore, Singapore

## Abstract

**Background:**

A characteristic difference between highly and non-highly pathogenic avian influenza strains is the presence of an extended, often multibasic, cleavage motif insertion in the hemagglutinin protein. Such motif is found in H7N3 strains from chicken farm outbreaks in 2012 in Mexico.

**Methods:**

Through phylogenetic, sequence and structural analysis, we try to shed light on the role, prevalence, likelihood of appearance and origin of the inserted cleavage motifs in these H7N3 avian influenza strains.

**Results:**

The H7N3 avian influenza strain which caused outbreaks in chicken farms in June/July 2012 in Mexico has a new extended cleavage site which is the likely reason for its high pathogenicity in these birds. This cleavage site appears to have been naturally acquired and was not present in the closest low pathogenic precursors. Structural modeling shows that insertion of a productive cleavage site is quite flexible to accept insertions of different length and with sequences from different possible origins. Different from recent cleavage site insertions, the origin of the insert here is not from the viral genome but from host 28S ribosomal RNA (rRNA) instead. This is a novelty for a natural acquisition as a similar insertion has so far only been observed in a laboratory strain before. Given the abundance of viral and host RNA in infected cells, the acquisition of a pathogenicity-enhancing extended cleavage site through a similar route by other low-pathogenic avian strains in future does not seem unlikely. Important for surveillance of these H7N3 strains, the structural sites known to enhance mammalian airborne transmission are dominated by the characteristic avian residues and the risk of human to human transmission should currently be low but should be monitored for future changes accordingly.

**Conclusions:**

This highly pathogenic H7N3 avian influenza strain acquired a novel extended cleavage site which likely originated from recombination with 28S rRNA from the avian host. Notably, this new virus can infect humans but currently lacks critical host receptor adaptations that would facilitate human to human transmission.

## Background

Influenza viruses are classified into 3 different types (A,B,C) and influenza A is further divided into specific subtypes named after the respective combination of surface protein variants pairing 1 of 17 hemagglutinins (the “H” in HxNx) with 1 of 10 neuraminidases (the “N” in HxNx). These subtypes are known to circulate preferably in specific bird species which possess sialic acid linked to oligosaccharides via alpha (2,3) linkages, such as chickens, turkeys, and ducks. [1,2]. There has been a recent outbreak of a new H7N3 strain in chicken farms in Mexico in June/July 2012, characterized as a highly pathogenic avian influenza (HPAI) strain [3]. While the epidemiological and initial genetic characterization of this outbreak strain has been described elsewhere [4,5], we would like to add information on the detailed origin of the extended cleavage site possibly responsible for making the strain highly pathogenic. The hemagglutinin cleavage site in the influenza A HA0 precursor protein typically contains a monobasic cleavage site with the consensus motif Q/E-x-R, allowing for cleavage of the HA after the “R”, usually by trypsin, into the HA1 and HA2 proteins [6]. In highly pathogenic avian influenza (HPAI) viruses, the HA0 cleavage site usually contains a multibasic cleavage site (MBCS) corresponding to a canonical R-x-K/R-R motif, suggesting that this motif is at least partially involved in the increased pathogenicity of the given HPAI strain. However, in some HPAI strains, in place of an MBCS, observations have been made of an extended cleavage site with multiple basic residues at positions other than the canonical site, which usually conform to the minimal R-x-x-R cleavage motif. Such motif differences can still result in functional cleavage sites, possibly changing the range of proteases or the same protease with different efficiencies. Gain of function of cleavability by ubiquitously expressed proteases opens the door for systemic replication of the virus and consequently increased pathogenicity [7]. Of particular interest is the situation of the inserted extended cleavage site (PENPK-**DRKSRHRR**TR/GLF, insertion in bold) in HA of A/chicken/Jalisco/CPA1/2012(H7N3). Firstly, it turns the classical monobasic cleavage motif into an extended RxxR cleavage site which could be targeted by an increased range of proteases, including matriptase among others. Secondly, with a register shift of two positions in N-terminal direction, there is also a canonical multibasic cleavage motif (RHRR = R-x-K/R-R) which could be hypothesized to be cleavable by furin or other subtilisin-like proteases. Multibasic cleavage sites (MBCS) in the influenza A hemagglutinin protein have been studied extensively in the context of pathogenicity in different viruses [6-8]. However, only H5 and H7 subtypes have been known to naturally acquire MBCSs, and this acquisition has been attributed to 2 distinct mechanisms, either by the random insertion or gradual accumulation of basic amino acids through mutations [9,10], or by recombination either with viral or host RNA [11,12], a phenomenon which has only been observed in H7 strains [11]. While the insertion of an MBCS is sufficient to turn low pathogenicity strains (LPAI) into high pathogenicity strains (HPAI) in chickens [13,14], this pathogenicity increase is not consistently observed in other poultry species such as ducks. This suggests that the acquisition of an MBCS is not the only pathogenicity determinant in these species – indeed, there are physiological differences between ducks and chickens, such as the lack of RIG-I in chickens, as well as differences in the upregulation of pro-inflammatory cytokines and interferons in response to HPAI infection [15]. Moreover, the increase of pathogenicity does not seem to translate directly to mammalian systems [16,17]. However, MBCS acquisition has been seen to alter the route for systemic infections [18]. Here, we used phylogenetic, sequence and structural analysis to shed light on the origin of the extended cleavage motif in this H7N3 avian influenza strain from recombination with host 28S rRNA as well as evaluate its potential for human-to-human transmission.

## Results and discussion

**Figure 1:**
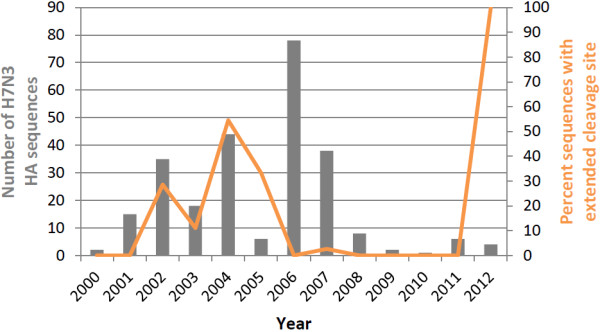
**Frequency of occurrence of H7N3 strains with and without extended cleavage site.** 257 HAs from H7N3 strains since the year 2000 were downloaded from GISAID and their absolute numbers per year shown as bar plot (values on left axis). The orange curve shows the percentage of sequences with extended cleavage site as defined by a consensus motif of R-x-x-R (where the final R is the normal cleavage site without insertion).

First, we investigated the frequency of extended cleavage site motifs of H7N3 strains in the EpiFlu database of the Global Initiative on Sharing All Influenza Data (GISAID) since the year 2000 (Figure 1). It can be seen that most H7N3 strains collected from 2006 to 2011 lack extended cleavage motifs with the consensus R-x-x-R, while in 2012 in the Mexican chicken farm outbreak sequences, we do see a prominent reappearance of such a motif.

Since the extended and multibasic cleavage sites are of importance for pathogenicity potential of influenza strains, we tried to shed further light on its possible origins. In the case of the previous occurrence of a highly pathogenic H7N3 in an outbreak in British Columbia in February 2004, it was found that the insertion resulting in the extended HA cleavage site was derived from intersegmental recombination from the matrix gene of the same virus [11]. Similarily, H7N3 strains from an outbreak in Chile in 2002 also had an extended cleavage site inserted through intersegmental recombination but from the viral NP gene instead [12]. Consequently, we tried to find if the extended cleavage site from the current Mexican H7N3 strains would have a similar intersegmental origin by comparing it to all segments of the virus using BLAST [19] with settings for small query sequences. However, as there was no significant hit in the genome of the virus itself we extended the search to all other known influenza viruses (potential for co-infection) and again did not find a hit. Next, we searched the nr/nt database [20] with restriction to chicken sequences and found a perfect match to a chicken 28S ribosomal RNA (rRNA) covering all 24 inserted nucleotides at 100% identity (E-value 4e-05). While the acquisition of an extended cleavage site by recombination has been rarely reported in comparison to random insertions of basic amino acids [11], having 28S rRNA as source for recombination is not surprising as it is an important molecule with high copy number in eukaryotes, including chicken [21]. Indeed, a similar insertion through recombination with host 28S rRNA has already been described previously [22] in an H7N3 lab strain from 1971 (…PENPKT-**SLSPLYPGRTTDLQVPTA**-R/GLF…, insertion bold between hyphens, classical cleavage site indicated by “/”). Although both insertions come from the 28S rRNA host gene, they are derived from different regions of the gene as can be seen by the different insertion sequence in the 2012 H7N3 strains (…PENPK-**DRKSRHRR**-TR/GLF…) and are certainly independent recombination events. While the 1971 insertion was only observed in a lab strain, it is noteworthy that the 2012 Mexican H7N3 acquisition of the extended cleavage site from host 28S rRNA appeared to have been the first observation of natural recombination of this kind.

Although the 28S rRNA origin of this 24 nucleotide sequence is unambiguous as the same sequence is not found in any other gene with the described searches, the exact genome mapping of 28S rRNA genes is tricky as they are encoded in repeated blocks in different copy numbers on different chromosomes with variation among individuals and, therefore, often omitted from reference genome assemblies. It has to be noted that due to the high conservation of the 28S rRNA, there are in principle also several other organisms including other birds, horses, pigs and even humans that share the same 100% identical fragment. Therefore, while it can be deduced that the insertion most likely originated from a eukaryotic host 28S rRNA, one cannot unambiguously identify this host, although an avian host appears the most likely scenario. Also, it is not possible to distinguish if the insertion first happened in the chicken farm or already before in wildbirds. Nevertheless, in this context it is interesting to note that some but not all chicken lines selected for enhanced growth or egg quality, size and number appear to have an increased rRNA gene copy number [23].

Considering the mechanism of recombination, previous studies [11,12,24] tried to find palindromic sequences at the junction of the insert to strengthen the possibility of RNA recombination but only for the 1971 insertion such motif was reported [22]. We reanalyzed previous and the current instances of cleavage site insertions for H7 strains and found previously undetected palindromic sequences at regions surrounding the respective inserts. For most of the palindromic sequences in Table 1, the midpoint of the pair of palindromic sequences occurs either exactly or in the vicinity from the start of the insert, thereby providing a plausible explanation as to how insertion might occur at the given site. While we also find a candidate palindromic recombination motif for the 2012 Mexican sequence, we acknowledge that this motif is short which increases the chance of random occurrence and not ideally positioned relative to the insertion site. Consequently, the exact mechanism of recombination for this particular insert remains to be elucidated.

**Table 1:** Palindromic sequences in the region surrounding the cleavage site insert provide insights to possible RNA recombination events

**Strain (Origin)**	**Sequence**
(Chicken 28S rRNA)	TGGTTCGATT***AGTCTTT***CGCCCCTATACCCGGGTCGGACGACCGATTTGCACGTCAGGACCGCTACG
A/turkey/Oregon/71	TCC***AAAGACT***AGTCTTTCGCCCCTATACCCGGGTCGGACGACCGATTTGCACGTCAGGACCGCTAGA
A/Seal/Mass/1/80 (NP)	AGAGGAGAAACAAATATCTGGAAGAGCATCCCAGTG***CTGGGA***AAGATCCTAAGAAGACAGGGGGTCCAATCTACAGGAGGAGAG
A/Seal/Mass/1/80	ATG***TCCCAG***AGAATCCAAAGAAAGAGCATCCCAGTGCTGGGAAAGATCCTAAGAAGACAGGGGGTCCAATCTACAGGAGGACCA
A/chicken/Chile/184240-1/2002 (NP)	TCAGCAGGACAGATAAGCGTGCAGCCCAC***TTTCT***CGGTGCAGAGAAACCTTC
A/chicken/Chile/184240-1/2002	TCC***AGAAA***AACCAAAGACATGCAGCCCACTTTCTCGGTGCAGAGAAACCAGA
A/chicken/BC/CN7/2004 (M1)	ATAATCTTCTTGAAAATTTGCAGGCCTACCAGAAACGAA***TGGGA***GTG
A/chicken/BC/CN7/2004	ACG***TCCCA***GAGAACCCCAAGCAGGCCTACCGGAAACGAATGACCAGA
(Chicken 28S rRNA)	CTTGGTGAATTCTGCTTCACAATGATAGGAAGAGCCGACATCGAAGGATCAAAAAGC***GACGT***CGC
A/chicken/Jalisco/CPA1/2012	GAA***ACGTC***CCAGAGAACCCCAAGGATAGGAAGAGCCGACATCGAAGGACCAGAGGCCTTTTTGGA

Representative H7 strains with known or predicted insert origin are presented in the table. The inserts (underlined) and 25 bases flanking the insert were examined for palindromic sequences together with the gene containing the putative insert origin. Data is presented as double row pairs where the top row represents the putative insert origin while the lower row represents the HA sequence with the observed insert. Palindromic sequences are represented in bold and italics.

**Figure 2:**
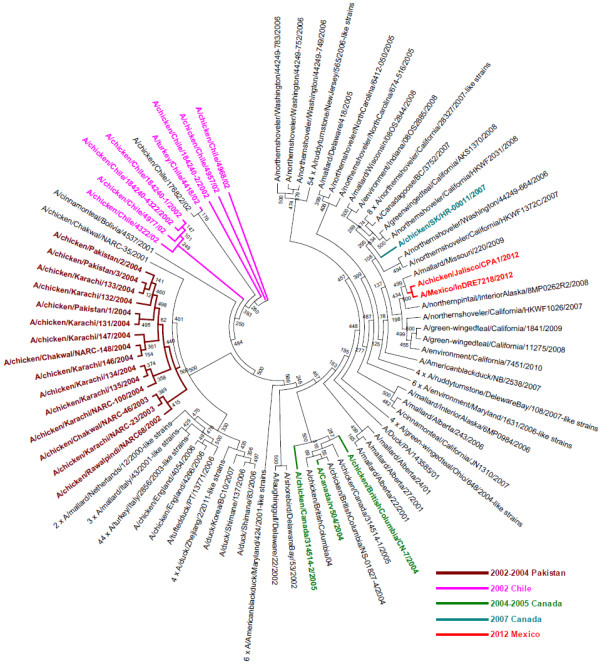
**Phylogenetic relationship between 205 H7N3 full-length HA nucleotide sequences collected from 2000 till 2012.** Strains with extended cleavage sites representing high pathogenic avian influenza (HPAI) strains as reported in the literature were separated by geographical regions and different years of outbreak and were highlighted with 5 different colors indicated in the legend. The recent 2012 Mexican chicken farm outbreak arose from a cluster of originally low pathogenic strains and therefore independently acquired the extended cleavage site which likely switched the strain to highly pathogenic.

In order to investigate if the extended cleavage site was also present in possible phylogenetic precursor strains, we analyzed the relation of H7N3 hemagglutinin sequences from 2000 until 2012. The phylogenetic tree (Figure 2) shows a scattered pattern of strains with and without the generic R-x-x-R cleavage site motifs and appearance of the current 2012 Mexican motif cannot be explained by descendance from an old strain that already had the insertion but, instead, it is missing in the genetically most closely related strains from the preceding years. Therefore, this extended cleavage site appears to be a recent acquisition which is consistent with earlier analysis of H7 evolution and lineages [1,25]. Looking at the hemagglutinin (HA) nucleotide sequences, the closest phylogenetic relatives with sequences in GISAID match the geotemporal context of occurrence in wild birds in southern states of the US in the preceding years, e.g. A/mallard/Missouri/220/2009(H7N3). Interestingly, when including all H7 subtypes into the analysis (Additional file 1: Figure S1), reassortment history of the ancestral H7 of the current H7N3 strains also includes recent combinations with N5 and N7, e.g. A/mallard/California/1390/2010(H7N5) and A/northernshoveler/Mississippi/09OS643/2009(H7N7). Therefore, a future detailed analysis of the reassortment history of these strains including all segments would be of interest. It should be noted that also the other close relatives from different H7Nx subtypes did not have the additional cleavage motif insertion.

**Figure 3:**
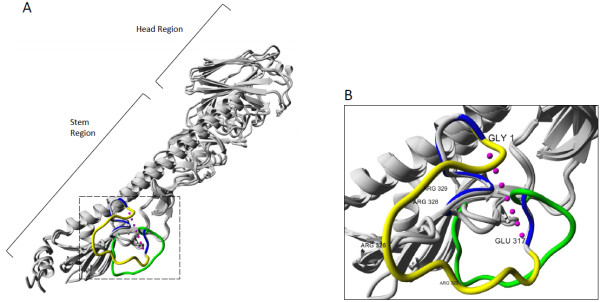
**Structure of the Influenza A hemagglutinin monomer showing a representative extended cleavage site. A**) The crystal structure representation of an uncleaved hemagglutinin HA0 precursor from subtype H3 (PDBID: 1HA0 [6], cleavage region green) overlaid with a structure of a cleaved HA1/HA2 heterodimer from a highly pathogenic H7 virus (PDBID: 4DJ6 [40], cleavage region blue) and an energy-minimized average representation of the cleavage loop for the highly pathogenic H7 with the new insertion modelled in YASARA Structure (yellow). Cleavage site indicated with dashed box at the stem region. **B**) Detailed structure of hemagglutinin cleavage site. Uncleaved loop (PDBID: 1HA0, green) shown with cleaved ends (PDBID: 4DJ6, blue) Glu317 of HA1 and Gly1 of HA2 connected with pink dots representative of the cleaved loop fragment. Basic arginine residues at positions 323, 326, 328 and 329 in the loop model are indicated.

The structural position of the inserted cleavage site is in the HA stem and away from the head region where functionally important host receptor and antibody binding sites are located (Figure 3). On the other hand, cleavage at this HA stem site is required for conformational changes allowing entry of the virus [6]. The assumed biomolecular mechanism of the increased pathogenicity in chickens through an extended cleavage motif is a gain of a trypsin-independent cleavage site which increases cleavage efficiency through utilizing ubiquitous proteases such as furin and other subtilisin-like proteases allowing infection of more cell types and tissues [7,26,27]. We show in a representative structural model of the hemagglutinin from this H7N3 strain using Yasara Structure [28] that the newly inserted cleavage site is, as expected, at the protein surface and accessible for protease cleavage (Figure 3). As seen in the crystal structure of furin with a substrate analog [29], the substrate cleavage motif structure appears linear and this is in agreement with linearly extended conformations accessible to the loop region as exemplified in the model. Furthermore, the dynamic structure of the insertion loop also suggests that mainly the relative but not absolute position of the positive charges for the new cleavage motif seems restricted and that it could flexibly accommodate different arrangements of related cleavage motifs of different length and sequence origin which increases the likelihood of insertion of a productive cleavage site and, hence, facilitates extended cleavage site occurrence. In addition to this, examination of the entire insert in the modified cleavage site using the ProP 1.0 furin cleavage predictor [30] has identified both the original RRTR motif at the P4-P1 positions, which conforms to the minimal requirement (R-x-x-R) for furin cleavage [31] as well as RHRR at the P5-P2 positions, which conforms to the canonical R-x-K/R-R furin cleavage site, as predicted furin substrates. Given the presence of these 2 motifs in such close proximity, and previous estimations of a log-difference in affinity between the canonical and minimal furin cleavage motifs [31], it is quite likely that the RHRR in the extended cleavage site would be preferentially bound in comparison to the existing monobasic RRTR cleavage site, allowing for non-specific cleavage by ubiquitous proteases.

The 2012 motif’s uniform presence in the outbreak sequences indicates that it must have quickly replaced any precursor without motif indicating a possible advantage for the new virus through the additional cleavage site, although this will have to be confirmed experimentally. In the case of the related H5N1 avian influenza viruses, there has been some increase in measures of severity in mice and ferrets through addition of an MBCS motif while there has been a lack of increased pathogenicity in non-human primate hosts [17]. In principle, H7 viruses have the potential to infect humans [2] and there have indeed been two human cases linked to the recent Mexican H7N3 outbreak [5]. Both cases recovered fully and only had mild symptoms, such as conjunctivitis. In this context it is important to note that animal to human influenza transmissions are rare and most often limited to close contact with the respective animals [32] as was also the case for the human H7N3 infections in Mexico [5].

Recently, 7 candidate positions have been identified where sets of 4 to 5 mutations allowed airborne transmission between ferrets of influenza viruses with an avian-derived hemagglutinin [33-35]. As the ferret setup serves as model for human to human transmission, we investigated the status of these key structural positions for transmission in the current H7N3 virus (including the human infection case with available HA virus sequence: A/Mexico/InDRE7218/2012(H7N3)). The human and chicken derived strains are identical at these positions and only one out of 7 candidate positions has the mammal-adapted residue while the others show the typically avian-cell preferring residues (Table 2). Since a critical number of 4 or more such adaptive mutations would be necessary to facilitate mammalian transmission [34], the risk for human-to-human transmissions of the current strain should be low.

**Table 2:** Comparison of residues at key sites shown to be important for potential human-to-human transmission

**Residue position**	**Wildtype residue in H5**	**Adaptation mutant residue in H5**	**Observed residue, 2012 H7N3**
**110 (103)**	H	Y	Q
**160 (156)**	T	A	A*
**158 (154)**	N	D	N
**224 (220)**	N	K	N
**226 (222)**	Q	L	Q
**228 (224)**	G	S	G
**318 (315)**	T	I	T

In order to examine the likelihood of efficient airborne human-to-human transmission of the 2012 H7N3 viruses, wildtype and mutant residues at previously identified key positions making H5 viruses transmissable in ferrets [33,35] were compared to the corresponding residues in the hemagglutinin amino acid sequence for H7N3. Listed residue positions are based on H3 numbering as used in Imai et al. followed by alternative numbering as in Herfst et al. in parenthesis. An asterisk * indicates the single position where the new strain has a mammalian transmission-adapted rather than the avian wildtype residue.

## Conclusions

This H7N3 outbreak strain is of special interest as an extended cleavage site including a shifted multibasic cleavage site has been newly acquired by the virus with likely origin from host 28S rRNA. We discuss that structural insertion of a productive cleavage site is quite flexible to accept insertions of different length and with sequences from different possible origins. Given the abundance of viral and certain host RNA in infected cells, the acquisition of a pathogenicity-enhancing extended cleavage site through a similar route by other low-pathogenic avian strains is possible, although other mechanisms of basic residue introduction through mutation proximal to the hemagglutinin cleavage site may be more common. Importantly, although this virus may be highly pathogenic in chickens, the few reported cases of human infections seemed to have had only mild symptoms and the structural sites known to enhance mammalian airborne transmission currently are dominated by the characteristic avian residues, so the risk for human-to-human transmission is low. Nevertheless, these positions should continue to be monitored if this strain continues to cause outbreaks in birds or even further human infections.

## Methods

### Extended cleavage site motif definition and determination of pathogenicity

In this study, the extended cleavage is defined by the consensus motif of R-x-x-R (where the final R is the normal cleavage site without insertion). The R-x-x-R sequence motif was used instead of the canonical R-x-R/K-R (commonly referred to as multibasic cleavage site) because there are examples of confirmed highly pathogenic strains that do not match the restrictive R-x-R/K-R pattern (for example A/chicken/Chile/2002 with motif R-E-T-R and A/chicken/BC/2004 with motif R-M-T-R, see Additional file 2: Table S1). Hence, the more general R-x-x-R motif has been used in our analyses. It should be noted that this did not increase the number of strains classified as HPAI except for including the Chile and BC strains with the degenerate motifs. Consequently, the large majority of strains analyzed here as having an extended cleavage site also conform to the canonical multibasic cleavage site. At the same time, MBCS or extended cleavage motif presence does not guarantee increased HA cleavability. Similarly, increased cleavability can, but also not necessarily has to, result in increased pathogenicity. Due to its importance, the endpoint of low and high pathogenicity is analyzed more often compared to HA cleavability directly and the experimental test of low or high pathogenicity as reported in the literature is hence used in this work as indirect evidence for increased cleavability of the observed motifs. Therefore, all H7Nx sequences that were found to contain the extended R-x-x-R motif (Additional file 2: Table S1) were traced to the source literature of their outbreak where they were distinguished as either LPAI strains or HPAI strains by the intravenous pathogenicity index in chickens as reported in the literature.

### Sequence data

257 HAs from H7N3 strains since the year 2000 with protein sequence information around the cleavage site were downloaded from the EpiFlu database of the Global Initiative on Sharing All Influenza Data (GISAID) and used to count the occurrence of extended cleavage sites in recent H7N3 sequences (Figure 1). For the phylogenetic analysis (Figure 2), 205 isolates (a subset of the 257 above) with complete HA nucleotide sequences were used. Another phylogenetic analysis was conducted on 1032 H7Nx strains using complete HA nucleotide sequences since the earliest available H7 sequence in GISAID in the year 1902. Files with the complete list of isolates and acknowledgment of submitting laboratories are available in Additional files 3 and 4 from the journal website.

*BLAST search:* The origin of the cleavage site inserts with length of 16 bases or more were derived by searching against the chicken reference genome using the NCBI megablast [19]. Next, the inserts were searched against the NCBI non-redundant database limited to chicken taxid for the best hit. The best hit was subsequently searched against the chicken reference genome to ensure that the insert and the predicted gene (also the best blast hit) maps to the same genomic location. The inserts were also searched against the non-redundant database limited to bird taxid and mammal taxid, and against influenza viruses.

### Search for palindromic sequences in vicinity of the insert

The nucleotide sequences of the predicted origin of the insert were aligned with representative H7 HA sequences. 25 bases flanking the insert were searched for palindromic sequences.

### Phylogenetic analyses

To examine the relationship between recent H7N3 outbreaks, 205 H7N3 full-length HA nucleotide sequences (collection date from 2000 till 2012) were aligned with MAFFT [36]*.* Next, a maximum likelihood tree was constructed using PHYML [37] with bootstrap test (500 steps), the HKY85 substitution model with gamma distribution (4 categories) and shape parameter (0.372) estimated by the program. The tree is displayed and colored in MEGA [38]. In order to understand the phylogeny of the 2012 Mexican strains (Additional file 1: Figure S1), 1032 H7Nx full-length HA nucleotide sequences (collection date from 1902 till 2012) were similarly aligned with MAFFT [36] and a neighbour joining tree using the Tamura-Nei model with gamma distribution (5 categories) was generated with MEGA [38].

### Structural modeling

The HA structure with cleavage loop for the highly pathogenic H7N3 strain was modelled in YASARA using the homology modelling procedure used in the CASP competition which has been shown to give accurate structures in the model refinement category [28]. The hemagglutinin sequence from A/chicken/Jalisco/Jal0612/2012 was used as a target and HA monomers from 2 subtype H7 structures (PDBID: 3M5G [39] and PDBID: 4DJ6 [40]) served as templates. It is important to note that the loop is flexible and can take up multiple conformations which are however constrained by the fixed endpoints and similar to each other and the minimized average conformation is shown in the Figure.

## Abbreviations

HA: Hemagglutinin; MBCS: Multibasic cleavage site; rRNA: Ribosomal RNA.

## Competing interests

The authors declare no competing interests.

## Authors’ contributions

SMS and FE conceived of the study. RTCL and SMS analyzed the motif occurrence and insertion origin. RTCL contributed the phylogenetic analysis. VG and SMS carried out the structural modeling. RTCL, VG, SMS and FE wrote parts of the manuscript. All authors read and approved the final manuscript.

## Supplementary Material

Additional file 1 **We acknowledge the authors, originating and submitting laboratories of the sequences from GISAID’s EpiFlu™ Database on which this research is based.** The list of submitters of H7N3 isolates used for analysis in Figures 1 and 2 is detailed below. Click here for file

Additional file 2 **We acknowledge the authors, originating and submitting laboratories of the sequences from GISAID’s EpiFlu™ Database on which this research is based.** The list of submitters of H7Nx isolates used for analysis in Figure S1 is detailed below. Click here for file

Additional file 3 Phylogenetic tree of the 2012 Mexican strains with 1032 H7Nx full-length HA nucleotide sequences. Click here for file

Additional file 4: Table S1 Comparison of H7 HA sequences containing inserts at the cleavage site. H7 sequences from GISAID were screened for the R-x-x-R consensus motif where the final R is the normal cleavage site without insertion. Each row in the table is a representative strain that contains a distinctive insert sorted by date. The amino acid sequences at the extended cleavage site are coloured based on their similarity and origin, with the same colouring scheme as the phylogenetic tree in supplementary Figure 1, while the inserted amino acids are in bold. The pathotype of the strains was inferred from literature reports. Click here for file
