# Supplementary material for: The highly pathogenic H7N3 avian influenza strain from July 2012 in Mexico acquired an extended cleavage site through recombination with host 28S rRNA
Source: Virol J. 2013 May 1;10:139. doi: 10.1186/1743-422X-10-139 (PMC3673898; doi:10.1186/1743-422X-10-139)
Supplement: Additional file 1 — We acknowledge the authors, originating and submitting laboratories of the sequences from GISAID’s EpiFlu™ Database on which this research is based. The list of submitters of H7N3 isolates used for analysis in Figures 1 and 2 is detailed below. [file 1743-422X-10-139-S1.pdf]

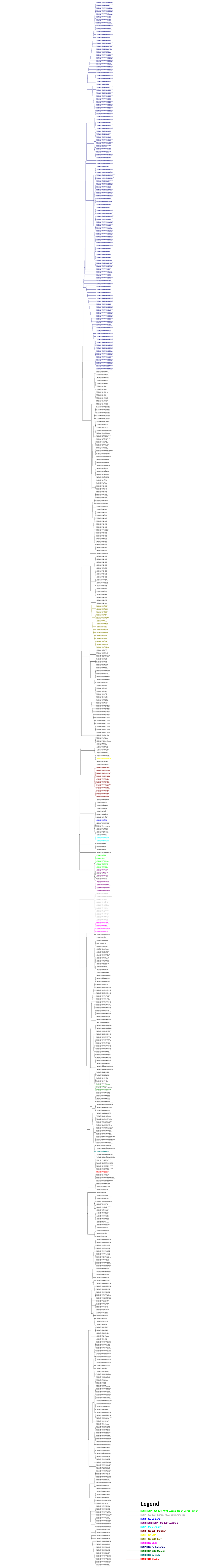
Legend
H7N1 H7N7 1901-1945 1993 Europe Japan Egypt Taiwan
H7N7 1956-1977 Europe USA SouthAmerica
H7N3 1963 England
H7N3 H7N4 H7N7 1976-1997 Australia
H7N7 1979 Germany
H7N3 1995-2004 Pakistan
H7N1 1994 USA
H7N1 1999-2000 Italy
H7N3 2002 Chile
H7N7 2003 Netherlands
H7N3 2004-2005 Canada
H7N3 2007 Canada
H7N3 2012 Mexico
