# Supplementary material for: The highly pathogenic H7N3 avian influenza strain from July 2012 in Mexico acquired an extended cleavage site through recombination with host 28S rRNA
Source: Virol J. 2013 May 1;10:139. doi: 10.1186/1743-422X-10-139 (PMC3673898; doi:10.1186/1743-422X-10-139)
Supplement: Additional file 2 — We acknowledge the authors, originating and submitting laboratories of the sequences from GISAID’s EpiFlu™ Database on which this research is based. The list of submitters of H7Nx isolates used for analysis in Figure S1 is detailed below. [file 1743-422X-10-139-S2.docx]

| **Years of Outbreak** | **Isolate^@^** | **Subtype** | **Region** | **Origin of insert** | **Amino acid sequence** | **Pathotype (L/H)** | **Reference** |
| --- | --- | --- | --- | --- | --- | --- | --- |
|  |  |  |  |  | PENPKTR/GLF | L |  |
|  |  | H5N1 |  |  | PQRERRRRKR/GLF |  |  |
| 1901-1902 | A/chicken/Brescia/1902 | H7N7 | Italy | - | PELPKK**RRK**R/GLF | H | http://www.ncbi.nlm.nih.gov/pubmed/11148006 |
| 1925 | A/chicken/Japan/1925 | H7N7 | Japan | - | PEIPKK**REK**R/GLF | H | http://www.ncbi.nlm.nih.gov/pubmed/16642763 |
| 1927-1933 | A/FPV/Dutch/27 | H7N7 | Europe | - | PELPKK**RRK**R/GLF | H | http://www.ncbi.nlm.nih.gov/pubmed/19618616 |
| 1933-1934 | A/chicken/Germany/1934 | H7N1 | Germany | - | PEPSKK**RKK**R/GLF | H | http://www.ncbi.nlm.nih.gov/pubmed/19618622 |
| 1945 | A/FPV/Egypt/45 | H7N1 | Egypt | - | PEFSKK**RRK**R/GLF | H | http://www.ncbi.nlm.nih.gov/pubmed/8503786 |
| 1956-1963 | A/equine/Prague-1/1956 | H7N7 | Czechoslovakia |  | PEAPAH**KQLTHHMRKK**R/GLF | H | http://www.ncbi.nlm.nih.gov/pubmed/13533033 |
| 1963 | A/turkey/England/1963 | H7N3 | England | - | PETPKR**RR**R/GLF | H | http://www.who.int/csr/don/2004_03_02/en/ |
| 1964-1977 | A/equine/Detroit/1/64 | H7N7 | USA/Europe/SouthAmerica |  | PENSTH**KQLTHHMRKK**R/GLF | H | http://www.ncbi.nlm.nih.gov/pubmed/14153655 |
| 1971 | A/turkey/Oregon/71* | H7N3 | - | 28S rRNA | PENPKT**SLSPLYPGRTTDLQVPTA**R/GLF | H | http://www.ncbi.nlm.nih.gov/pubmed/2544809 |
| 1976-1985 | A/chicken/Victoria/1/1985 | H7N7 | Australia | - | PEIPKK**REK**R/GLF | H | http://www.who.int/csr/don/2004_03_02/en/ |
| 1979 | A/chicken/Leipzig/79 | H7N7 | Germany | - | PEIPKK**KK**R/GLF | H | http://www.who.int/csr/don/2004_03_02/en/ |
| 1980 | A/Seal/Mass/1/80* | H7N7 | - | NP | PENPK**KEHPSAGKDPKKTGGPIYRR**TR/GLF | H | http://www.ncbi.nlm.nih.gov/pubmed/8091680 |
| 1992-1995 | A/chicken/Victoria/1/92 | H7N3 | Australia | - | PEIPKK**KK**R/GLF | H | http://www.who.int/csr/don/2004_03_02/en/ |
| 1995-2004 | A/chicken/Pakistan/447/95 | H7N3 | Pakistan | - | PETPKR**KRK**R/GLF | H | http://www.who.int/csr/don/2004_03_02/en/ |
| 1993 | A/duck/Taiwan/33/1993 | H7N7 | Taiwan | - | PEIPKK**REK**R/GLF | H^ |  |
| 1994 | A/Pekinrobin/California/30412/1994 | H7N1 | USA | - | PEIPKR**R**R/GLF | H | http://www.ncbi.nlm.nih.gov/pubmed/21900520 |
| 1997 | A/chicken/NSW/1/1997 | H7N4 | Australia | - | PEIPRK**RK**R/GLF | H | http://www.who.int/csr/don/2004_03_02/en/ |
| 1999-2000 | A/chicken/Italy/1082/1999 | H7N1 | Italy | - | PEIPKG**SRVR**R/GLF | H | http://www.who.int/csr/don/2004_03_02/en/ |
| 2002 | A/chicken/Chile/2002 | H7N3 | South America | NP | PEKPKT**CSPLSRCRET**R/GLF | H | http://www.ncbi.nlm.nih.gov/pubmed/15200862 |
| 2003 | A/Netherlands/065/03 | H7N7 | Netherlands | - | PEIPKR**RR**R/GLF | H | http://www.who.int/csr/don/2004_03_02/en/ |
| 2004-2005 | A/chicken/BC/2004 | H7N3 | Canada | M1 | PENPK**QAYQKRM**TR/GLF | H | http://www.ncbi.nlm.nih.gov/pubmed/15722533 |
| 2007 | A/chicken/SK-HR-00011/2007 | H7N3 | Canada | 28S rRNA? | PENPKT**TKPRPR**R/GLF | H | http://www.ncbi.nlm.nih.gov/pubmed/19788823 |
| 2008 | A/chicken/England/1158-11406/2008# | H7N7 | England | - | PEIPKR**KK**R/GLF | H | http://www.ncbi.nlm.nih.gov/pubmed/20521631 |
| 2009 | A/chicken/Spain/6279-2/2009^#^ | H7N7 | Spain | 28S rRNA? | PELPKG**TKPRPR**R/GLF | H | OIE Update on highly pathogenic avian influenza in animals (type H5 and H7), REF OIE: 8521. |
| 2012 | A/chicken/Jalisco/CPA1/2012 | H7N3 | Mexico | 28S rRNA | PENPK**DRKSRHRR**TR/GLF | H | OIE Update on highly pathogenic avian influenza in animals (type H5 and H7), REF OIE: 12067. |

^@^Name of an isolate that came from the year and geographical region of the outbreak.

?Origin of insert obtained from blast searches returned 28S rRNA as the best hit, with 100% sequence identity over 18 bases, and an E-value of 1.1. This has to be seen with caution as this E-value is not significant possibly due to the short length of the match.

*Lab strains.

^#^Incomplete sequences.

^Pathotype status inferred from A/chicken/Japan/1925.

**Supplementary Table 1. Comparison of H7 HA sequences containing inserts at the cleavage site.** H7 sequences from GISAID were screened for the R-x-x-R consensus motif where the final R is the normal cleavage site without insertion. Each row in the table is a representative strain that contains a distinctive insert sorted by date. The amino acid sequences at the extended cleavage site are coloured based on their similarity and origin, with the same colouring scheme as the phylogenetic tree in supplementary Fig. 1, while the inserted amino acids are in bold. The pathotype of the strains was inferred from literature reports.
